# Tracking SARS-CoV-2 Variants Using a Rapid Typification Strategy: A Key Tool for Early Detection and Spread Investigation of Omicron in Argentina

**DOI:** 10.3389/fmed.2022.851861

**Published:** 2022-05-17

**Authors:** Gonzalo M. Castro, Paola Sicilia, María Laura Bolzon, Laura Lopez, María Gabriela Barbás, María Belén Pisano, Viviana E. Ré

**Affiliations:** ^1^Laboratorio Central de la Provincia de Córdoba, Ministerio de Salud, Gobierno de la Provincia de Córdoba, Córdoba, Argentina; ^2^Área de Epidemiología, Ministerio de Salud, Gobierno de la Provincia de Córdoba, Córdoba, Argentina; ^3^Secretaría de Prevención y Promoción de la Salud, Ministerio de Salud, Gobierno de la Provincia de Córdoba, Córdoba, Argentina; ^4^Instituto de Virología “Dr. J. M. Vanella”, CONICET, Facultad de Ciencias Médicas, Universidad Nacional de Córdoba, Córdoba, Argentina

**Keywords:** SARS-CoV-2, variants of concern (VOCs), Omicron, diagnosis, real time RT-PCR, rapid screening

## Abstract

SARS-CoV-2 variants of concern (VOC) and interest (VOI) present mutations in reference to the original virus, being more transmissible. We implemented a rapid strategy for the screening of SARS-CoV-2 VOC/VOIs using real time RT-PCR and performed monitoring and surveillance of the variants in our region. Consecutive real-time RT-PCRs for detection of the relevant mutations/deletions present in the Spike protein in VOC/VOIs (TaqMan™ SARS-CoV-2 Mutation Panel, Applied Biosystems) were implemented. A total of 6,640 SARS-CoV-2 RNA samples (Cts < 30) from infected individuals in Central Argentina during 2021 were analyzed using different algorithms that were gradually adapted to the changing scenarios of local variant circulation. The strategy developed allowed the early detection and the identification of VOC/VOIs that circulated through the year, with a 100% of concordance with the WGS. The analyses of the samples showed introductions of VOCs Alpha and Gamma in February and March 2021, respectively. Gamma showed an exponential increase, with a peak of detection in July (72%), being responsible of the second wave of COVID19 in Argentina. Since VOC Delta entered into the region, it increased gradually, together with VOI Lambda, replacing VOC Gamma, until being the main variant (84.9%) on November. By December, these variants were replaced by the emergent VOC Omicron in a term of 2 weeks, producing the third wave. We report a useful tool for VOC/VOI detection, capable to quickly and cost-effectively monitor currently recognized variants in resource-limited settings, which allowed to track the recent expansion of Omicron in our region, and contributed to the implementation of public health measures to control the disease spread.

## Introduction

**FIGURE 1:**
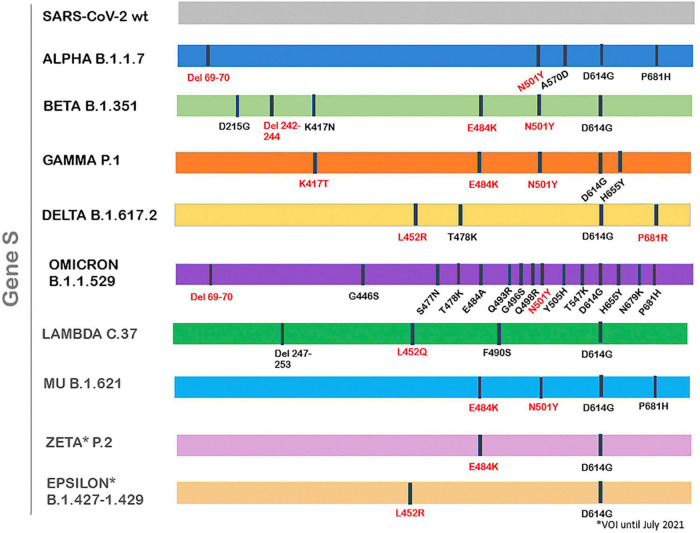
VOC/VOIs relevant mutations within the S gene.

Numerous severe acute respiratory syndrome coronavirus 2 (SARS-CoV-2) variants have already been documented globally during the COVID-19 pandemic. These viruses present one or more mutations in reference to the original virus, first isolated in Wuhan in 2019 (consensus sequence WIV04) (1, 2). Most nucleotide changes have little to no impact on the virus’ properties; however, there are mutations that produce phenotypic changes, which may affect virus transmissibility, severity, response to the vaccine or diagnostic tools (3). According to the virus’s features given by the mutations, the World Health Organization (WHO) and the Centers for Disease Control and Prevention (CDC) have classified some SARS-CoV-2 variants into variants of concern (VOC) and variants of interest (VOI) (3, 4). Thus, the first VOC described was the Alpha variant (lineage B.1.1.7), detected firstly in United Kingdom on September 2020, which rapidly expanded and become predominant in many countries, being the main variations the HV 69–70 deletion and N501Y mutation in the Spike protein (1, 5) (Figure 1). After that, VOCs Beta (lineage B.1.351) and Gamma (lineage P.1) were reported, first isolated in South Africa and Brazil, respectively, presenting high transmissibility rates too, and with the common mutation N501Y. Delta variant (lineage B.1.617.2) was detected for the first time in India in October 2020 and declared VOC on May 2021, displacing the rest of the VOCs in many parts of the world, with the presence of the main mutations L452R and P681R among others (6, 7). On November 2021, a new variant was detected in South Africa, containing numerous mutations/deletions in the Spike protein, which confer this variant more transmissibility. For this reason, it was declared VOC and named Omicron (lineage B.1.1.529) (2).

Over the last few months, several variants were classified as of interest (VOI), but later demonstrated to no longer pose a major added risk to global public health compared to other circulating SARS-CoV-2 variants, so they were re-classified as variants under monitoring (VUM) or even removed from the VOI/VUM list (2). At present, there are 2 VOIs: Lambda and Mu (2). Lambda (lineage C.37), first detected in Peru on December 2020, was declared VOI on June 2021, and presents the characteristic mutation L452Q. Mu (lineage B.1.621), first isolated in Colombia and declared VOI at the end of August 2021, presents the main mutations N501Y, E484K and P681H (2, 7). Former VOIs Zeta and Epsilon were declared of interest between March and July 2021 and present the mutations E484K and L452R, respectively (2, 7). Figure 1 shows VOC/VOIs relevant mutations in the S gene.

Due to the increased transmissibility, possible increased virulence or changes in clinical disease presentation, and immune escape (6), VOC/VOIs have the highest priority for surveillance, either to describe its circulation, map their spread, and to detect the introduction of new variants in a region. Currently, this surveillance is based on whole genome sequencing (WGS), an accurate method that generates detailed information, considered the gold standard technique to detect VOC/VOIs (5, 8). However, it is time-consuming, expensive, and requires trained staff and specific equipment, which restrict the access in resource-limited settings such as our region. So, it is not possible to apply this technique massively or to obtain results in a short-period time (5).

Since March 2020, in Argentina, and particularly in Córdoba Province, molecular surveillance of SARS-CoV-2 began with WGS (9). As a result of increased concern in public health due to the emergent variants, in the last months there has been a rapidly growing demand for WGS. In February 2021, the strategy of Sanger partial sequencing of the S gene was implemented complementary to WGS, for the search of VOC/VOIs in our region (10). However, both methods do not allow the rapid identification of VOCs (WGS demands at least 5 days for processing), delaying their spread control. Based on these facts, it was necessary to provide a rapid typing response, from the laboratory, in order to take measures at the public health level to contain the spread of Delta first, and Omicron later.

In this context, with limited resources, and in a particular scenario of changing viral circulation, including variants indigenous of Latin America (Gamma, Lambda, Mu), the objective of this work was to develop a strategy using real time RT-PCR for detection of VOC/VOI relevant mutations for molecular surveillance and rapid identification.

## Materials and Methods

### Samples

A total of 6,640 positive SARS-CoV-2 RNAs with Cts < 30, obtained from oropharyngeal swab samples from individuals from the province of Córdoba, Argentina (central region of the country), between 1st January and 31st December 2021 were analyzed to screen VOC and VOI mutations by real time RT-PCR. The samples had originally been extracted with MegaBio plus Virus RNA Purification Kit II (BioFlux) on the GenePure Pro Nucleic Acid Purification System NPA-32P and amplified by real time RT-PCR using DisCoVery SARS-CoV-2 Nucleic Acid Detection Kit.

### Detection of VOCs by Real Time PCR

TaqMan™ SARS-CoV-2 Mutation Panel (Applied Biosystems) was used for detection of the following relevant mutations/deletions present in VOC/VOIs (7), compared to the reference sequence WIV04 (wild type): HV 69–70 del, N501Y, E484K, L452R, K417T, P681R, 242–244 del, L452Q. Briefly, 7 μL of RNA were added to 8 μL of a mixture containing TaqPath™ 1-Step RT-qPCR Master Mix, CG (4X), TaqMan™ SARS-CoV-2 Mutation Panel Assay (40X) and nuclease-free water.

A strategy for detection of VOC/VOIs was implemented based on the different mutations found for each of the variants. Former VOIs Zeta and Epsilon were also included only in the samples from January to July, when those variants were classified as VOIs. The following samples were used as reference (obtained in collaboration with PAIS Project) (9): EPI_ISL_2271687 to EPI_ISL_2271690 (VOC Gamma), EPI_ISL_2007514 to EPI_ISL_2007516 (VOC Alpha), EPI_ISL_3230016 to EPI_ISL_3230018 (VOC Delta), EPI_ISL_8207589 to EPI_ISL_8207591 (VOC Omicron), EPI_ISL_3183944 (former VOI Epsilon), EPI_ISL_3183946 (VOI Lambda), EPI_ISL_3183947 (former VOI Zeta), EPI_ISL_6032791 (VOI Mu).

## Results

**FIGURE 2:**
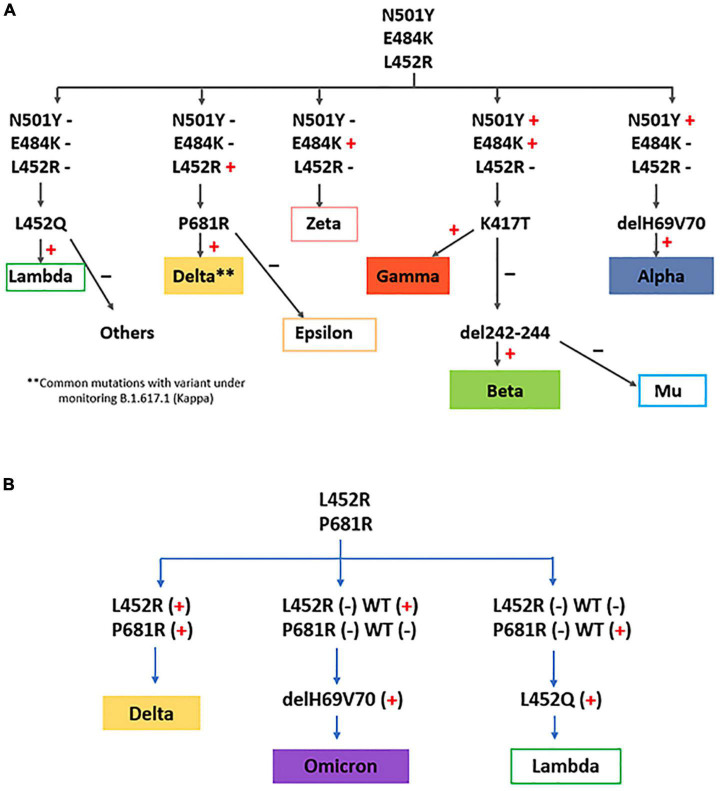
VOC/VOI detection strategies implemented in this study, using consecutive real time RT-PCRs for relevant mutation screening adapted to the different epidemiological scenarios. **(A)** algorithm used until October 2021. **(B)** algorithm used since November 2021.

We implemented several strategies for detection of SARS-CoV-2 VOCs (Alpha, Beta, Gamma, Delta, Omicron) and VOIs [Lambda and Mu; additionally, Zeta and Epsilon (VOIs until July 2021)] by real time RT-PCR looking for some characteristic mutations, depending on the epidemiological scenario. From January to October 2021, a first screening for N501Y, E484K and L452R mutations was carried out (Figure 2A). Based on the results, the following algorithm was continued:

a) N501Y(+), E484K(+), L452R(-): detection of K417T was performed; a positive result was indicative of VOC Gamma (N501Y, E484K, K417T). A negative result was subjected to detection of 242–244 del: if the deletion was present, VOC Beta would be the infecting variant (N501Y, E484K, 242–244 del). A negative result for K417T and 242–244 del, in the presence of mutations N501Y and E484K, was indicative of VOI Mu.
b) N501Y(+), E484K(-), L452R(-): deletion 69–70 was investigated; a positive result was indicative of VOC Alpha.
c) N501Y(-), E484K(+), L452R(-): the presence of only the E484K mutation would rule out the presence of VOCs, and would be indicative of former VOI Zeta.
d) N501Y(-), E484K(-), L452R(+): detections of P681R was performed; a positive result was indicative of possible VOC Delta. A negative result for P681R was indicative of former VOI Epsilon.
e) N501Y(-), E484K(-), L452R (-): detection of L452Q was performed; if this mutation was present, VOI Lambda would be the variant.

From November 2021, the strategy was changed, and a new combination of mutations for screening of VOC/VOIs was performed, based in the new scenario of variant circulation (high circulation of Delta, absence of VOC Alpha, little circulation of Gamma and Lambda, and imminent entry of Omicron) (Figure 2B). So, a first screening for L452R and P681R mutations was carried out, and the following algorithm was continued:

a) L452R (+), P681R (+): VOC Delta.
b) L452R (-), P681R (-): if position 452 was negative for wild type amplification, and position 681 was positive for wild type amplification, detection of L452Q was performed; if this mutation was present, VOI Lambda would be the variant.
c) L452R (-), P681R (-): if position 452 was positive for wild type amplification, and position 681 was negative for wild type amplification, detection of deletion 69–70 was investigated; a positive result was indicative of VOC Omicron.

**FIGURE 3:**
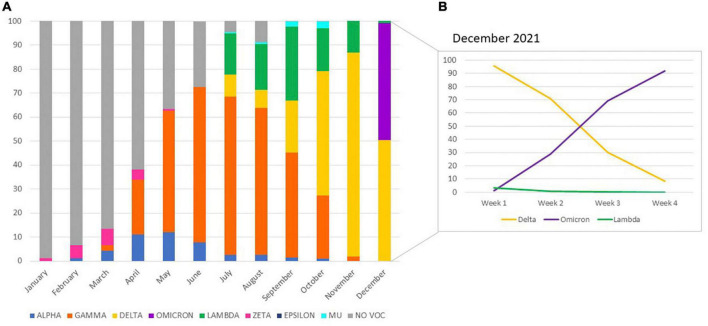
Distribution of SARS-CoV-2 variants of concern (VOC) and interest (VOI) detected in the community from Córdoba province, Argentina, by real time RT-PCR, January to December 2021 **(A)**. The frequency of variant detections performed on December are shown by week **(B)**.

With these strategies, a total of 6,640 samples was tested. Figure 2 shows changes in VOC/VOIs distribution within SARS-CoV-2 infections among the community in Córdoba during the studied period. VOCs Alpha and Gamma were first detected in our region in the months of February and March, respectively. From that moment, they began to circulate in the population. VOC Alpha was detected until October (0.9%), reaching a peak of 12.2% in May (Figure 3A). Since its first detection in March 2021, VOC Gamma presented an exponential rice, and became predominant in the following months, reaching a peak of detection on July (65.8%) (Figure 3A), being the main variant responsible of the COVID19 second wave in our region. From that moment, this VOC started to decrease, until November 2021, when the last detections occurred. VOC Beta was not detected.

VOC Delta was first detected in a traveler from Peru on July 2021, and from that moment it started to circulate in our region, increasing gradually, to reach its peak on November, with a frequency of 84.9%. From the first week from December, this variant started to decrease. VOC Omicron was first identified in two travelers, one from Dubai on December 6th, and the other from United States on December 12th. In 2 weeks, VOC Omicron became to be the predominant variant, displacing Delta and the other variants, with a current frequency of detection in the community of more than 90% (end of December 2021) (Figure 3B).

Regarding VOIs, Zeta circulated from January to May in a low frequency (1.1 to 0.7%, with a peak in March of 6.9%). Epsilon was only detected in August (0.1%). VOIs Lambda and Mu started to be screened on July 2021. Lambda was detected in the 17.3% of the samples that month, and it gradually increased its frequency until reaching 30.8% on September. From that moment, its frequency decreased until its complete absence at the end of December. VOI Mu was detected in a low frequency (less than 3%) from July to October.

## Discussion

The VOC/VOI detection strategy using consecutive real-time RT-PCRs for detection of relevant mutations implemented in our laboratory resulted a very useful and cost-effective diagnostic tool for the typing of variants. It was possible to monitor the variation of VOC/VOI distribution in the population over time, detecting changes in the variant circulation pattern, as well as to perform surveillance in travelers, which allowed an early detection of VOC Delta (making it possible to take measures to delay its spread) and VOC Omicron.

In Argentina, variant surveillance of SARS-CoV-2 and the study of variant spread is performed by national entities (National Ministry of Health and National Ministry of Science and Technology), using WGS or partial sequencing of the Spike protein, in a limited number of centers equipped with the necessary infrastructure. For this, positive traveler’s samples and randomly selected positive samples from each province are sent for processing to the laboratory in charge of carrying out the sequencing. The time between sending the samples and obtaining the result is between 2 and 15 days. Although WGS and partial sequencing provide accurate information (mainly WGS), they are laborious, time consuming, expensive and require extensive data processing, which has led to the search for faster and simpler alternatives for VOC/VOI detection (5, 8). In this sense, the strategy implemented during this work allowed to process a high number of samples, obtaining the typing result routinely. While the number of complete genomes from Córdoba province reached 460 during the studied period (9, 11), the samples processed by the real time RT-PCR for detection of VOC/VOI was 6,640. In addition, sample processing was considerably faster, without the need of sample derivation to the sequencing center; furthermore, costs were lower. In this way, Córdoba is the first province to implement this strategy for screening of VOC/VOI in Argentina, providing a quick and simple methodology that can be transferred to other laboratories that require it.

Using these real time RT-PCRs, it was possible to efficiently monitor variant circulation in the community since the introduction of VOC Alpha and Gamma in February and March 2021, respectively. Thus, in the following months, it was observed that, unlike the United States and Europe, VOC Gamma had an exponential increase and gained prominence over VOC Alpha and other variants, as happened in neighboring countries, such as Brazil and Chile (12), being responsible of the COVID19 second wave in our region. Moreover, results obtained showed concordance with those obtained by WGS (11).

On the other hand, the strategy developed during this study, which combines the detection of characteristic mutations of VOC/VOIs, made it possible to carry out different algorithms that gradually adapted to the scenario of local circulation of variants. Thus, it allowed a rapid identification of VOC Delta first, and Omicron then, key to take public health measures. The early detection of Delta allowed to isolate case zero and all its close contacts, circumscribing the outbreak and delaying the virus spread in our province. Subsequently, Delta began to be detected in the community, gradually increasing its proportion throughout the months studied. This coincided with the decrease in the number of SARS-CoV-2 positive cases (13), unlike what was observed in other parts of the world (4, 7), where the sustained advance of VOC Delta drove new waves of infections (14). Some reasons that could explain this difference include the exhaustive case identification, study and isolation of close contacts of positive Delta cases carried out by the Government of the Province, different epidemiological scenarios (Delta entered into our region with a particular variant circulation, different from Europe and United States, in which VOC Gamma presented the highest frequency), acquired immunity of the population associated with the second wave (occurred on May-July 2021 in our province), different vaccination coverages and different vaccination programs implemented in the countries.

Regarding VOC Omicron, Córdoba was the first province to detect its circulation in the community and describe its spread using a large-scale typing strategy. Tracking this VOC daily, an abrupt rise was observed, as described in other countries (3, 15), becoming the predominant VOC in 4 weeks, displacing Delta, and being responsible of the third wave in our region.

Former VOIs Zeta and Epsilon were detected in low percentages in the period studied, with currently no detection, supporting the new WHO classification, in which they are no longer designated as variants of interest or variants under surveillance (3).

VOI Lambda was the second major variant of circulation in the community throughout many of the months studied, coinciding with what was reported at the national and regional levels (7, 9, 11). However, since VOCs Delta and Omicron gained prominence, Lambda was no longer detected.

The strategy described here present some limitations: (a)-some samples show inconclusive results, (b)-samples with Ct values > 30 cannot be typed, (c)-many diagnostic PCR platforms can deplete swab material, leaving an inadequate volume of residual sample for a multitube mutation screen (16), (d)-since VOC/VOI classification is dynamic and is constantly changing (3), the strategy must be constantly reviewed and evaluated in order to corroborate whether the algorithm used is adequate for a correct VOC typing, (e)-it is not possible to find mutations other than those specifically searched for, which leads to not being able to detect new emerging VOC/VOIs. Some of these limitations could be overcome by performing some modifications to the protocol (nested-PCRs, partial sequencing, etc.), although these would be detrimental to the practicality of the developed strategy, which was the objective of this work. All the exposed show that WGS cannot be replaced by real time RT-PCR specific for VOC/VOI. Furthermore, these methodologies complement each other. While specific real time RT-PCRs for mutations of interest are a useful tool for rapid VOC/VOI screening, WGS-based parallel surveillance is critical to detect new emerging variants and to study phylogeographic relationships between circulating viruses.

In conclusion, we report a valid strategy based on real time RT-PCR for VOC/VOI detection, first implemented in Argentina, which balance cost and time processing, capable to monitor currently recognized variants of concern. In the present moment requiring rapid strain typing to guide public health measures, such a rapid and accessible approach is essential.

## Data Availability Statement

The raw data supporting the conclusions of this article will be made available by the authors, without undue reservation.

## Ethics Statement

Ethical review and approval was not required for the study on human participants in accordance with the local legislation and institutional requirements. Written informed consent for participation was not required for this study in accordance with the national legislation and the institutional requirements.

## Author Contributions

GMC and PS performed the conceptualization, designed the experiments, and performed the molecular reactions. MLB carried out the molecular reactions. LL investigated and analyzed the epidemiological data. MGB participated in the conceptualization and supervised the work. MBP participated in the conceptualization and design of the experiments, analyzed the data, and wrote the original draft. VER participated in the conceptualization and design of the experiments, analyzed the data, supervised the work, and provided critical revision of the article. All the authors contributed to the article and approved the submitted version.

## Conflict of Interest

The authors declare that the research was conducted in the absence of any commercial or financial relationships that could be construed as a potential conflict of interest.

## Publisher’s Note

All claims expressed in this article are solely those of the authors and do not necessarily represent those of their affiliated organizations, or those of the publisher, the editors and the reviewers. Any product that may be evaluated in this article, or claim that may be made by its manufacturer, is not guaranteed or endorsed by the publisher.

## References

[1] Janik E Niemcewicz M Podogrocki M Majsterek I Bijak M. The emerging concern and interest SARS-CoV-2 variants. *Pathogens.* (2021) 10:633. 10.3390/pathogens10060633 34064143 PMC8224338

[2] GISAID web site. *Official hCoV-19 Reference Sequence.* (2022). Available online at: https://www.gisaid.org/resources/hcov-19-reference-sequence/ (accessed January 3, 2022).

[3] World Health Organization [WHO]. *Tracking SARS-CoV-2 Variants.* (2022). Available online at: https://www.who.int/activities/tracking-SARS-CoV-2-variants/tracking-SARS-CoV-2-variants (accessed January 3, 2022).

[4] Centers for Disease Control and Prevention [CDC]. *SARS-CoV-2 Variant Classifications and Definitions.* (2022). Available online at: https://www.cdc.gov/coronavirus/2019-ncov/variants/variant-info.html (accessed January 3, 2022).

[5] Ong DSY Koeleman JGM Vaessen N Breijer S Paltansing S de Man P. Rapid screening method for the detection of SARS-CoV-2 variants of concern. *J Clin Vrol.* (2021) 141:104903. 10.1016/j.jcv.2021.104903 34182300 PMC8213512

[6] Boehm E Kronig I Neher RA Eckerle I Vetter P Kaiser L Novel SARS-CoV-2 variants: the pandemics within the pandemic. *Clin Microb Infect.* (2021) 27:1109–17. 10.1016/j.cmi.2021.05.022 34015535 PMC8127517

[7] Outbreak Info. *Variants of Concern Reports.* (2021). Available online at: https://outbreak.info/situation-reports (accessed January 4, 2021).

[8] Lind A Barlinn R Landaas ET Andresen LL Jakobsen K Fladeby C Rapid SARS-CoV-2 variant monitoring using PCR confirmed by whole genome sequencing in a high-volume diagnostic laboratory. *J Clin Virol.* (2021) 141:104906. 10.1016/j.jcv.2021.104906 34273860 PMC8262397

[9] PAIS Consortium. *PAIS Consortium: Proyecto Argentino Interinstitucional de genómica de SARS-CoV-2 (Consorcio proyecto PAIS).* (2022). Available online at: http://pais.qb.fcen.uba.ar/ (accessed January 4, 2022).

[10] Torres C Mojsiejczuk L Acuña D Alexay S Amadio A Aulicino P Cost-effective method to perform SARS-CoV-2 variant surveillance: detection of Alpha, Gamma, Lambda, Delta, Epsilon and Zeta in Argentina. *Front Med.* (2021) 8:755463. 10.3389/fmed.2021.755463 34957143 PMC8703000

[11] Ministerio de Salud de la Nación Argentina, Información Epidemiológica. *Informes de Vigilancia Genómica.* (2022). Available online at: https://www.argentina.gob.ar/coronavirus/informes-diarios/vigilancia-genomica (accessed January 4, 2022).

[12] Brazil Mutation Report, Chile Mutation Report. *Alaa Abdel Latif, Julia L. Andersen, Andrew I. Su, Karthik Gangavarapu, Laura D. Hughes, and the Center for Viral Systems Biology. outbreak.info, Brazil Mutation Report and Chile Mutation Report.* (2021). Available online at: https://outbreak.info/location-reports?loc=BRA (accessed August 13, 2021).

[13] Government of the Province of Cordoba. *Informe Diario de Casos y Medidas.* (2022). Availabe online at: https://www.cba.gov.ar/informe-diario-de-casos-y-medidas/?csrt=13034516782944616592 (accessed January 4, 2022).

[14] Torres C Debat H Viegas M. Biological characteristics of SARS-CoV-2 variants of epidemiological interest and their impact on vaccine efficacy and effectiveness. *Preprint.* (2021): 10.1590/SciELOPreprints.2886

[15] Our World in Data. (2022). *Our World in Data.* Available online at: https://ourworldindata.org/covid-vaccinations (accessed January 4, 2022).

[16] Wang H Jean S Eltringham R Madison J Snyder P Tu H Mutation-Specific SARS-CoV-2 PCR screen: rapid and accurate detection of variants of concern and the identification of a newly emerging variant with spike L452R mutation. *J Clin Microbiol.* (2021) 59:e0092621. 10.1128/JCM.00926-21 34011523 PMC8288299

[17] Castro GM Sicilia P Bolzon ML Lopez L Barbas MG Pisano MB Strategy for a rapid screening and surveillance of SARS-CoV-2 variants by real time RT-PCR: a key tool that allowed control and delay in Delta spread in Cordoba, Argentina. *medrxiv [preprint].* (2021): 10.1101/2021.11.16.21266265

